# Reduced Programming Time and Strong Symptom Control Even in Chronic Course Through Imaging-Based DBS Programming

**DOI:** 10.3389/fneur.2021.785529

**Published:** 2021-11-08

**Authors:** Florian Lange, Frank Steigerwald, Tobias Malzacher, Gregor Alexander Brandt, Thorsten Michael Odorfer, Jonas Roothans, Martin M. Reich, Patrick Fricke, Jens Volkmann, Cordula Matthies, Philipp D. Capetian

**Affiliations:** ^1^Department of Neurology, University Hospital and Julius-Maximilians-University, Wuerzburg, Germany; ^2^Department of Neurosurgery, University Hospital and Julius-Maximilians-University, Wuerzburg, Germany

**Keywords:** directional deep brain stimulation, image-guided programming, subthalamic nucleus, chronic stimulation, randomized controlled double-blind study, Parkinson's disease

## Abstract

**Objectives:** Deep brain stimulation (DBS) programming is based on clinical response testing. Our clinical pilot trial assessed the feasibility of image-guided programing using software depicting the lead location in a patient-specific anatomical model.

**Methods:** Parkinson's disease patients with subthalamic nucleus-DBS were randomly assigned to standard clinical-based programming (CBP) or anatomical-based (imaging-guided) programming (ABP) in an 8-week crossover trial. Programming characteristics and clinical outcomes were evaluated.

**Results:** In 10 patients, both programs led to similar motor symptom control (MDS-UPDRS III) after 4 weeks (medicationOFF/stimulationON; CPB: 18.27 ± 9.23; ABP: 18.37 ± 6.66). Stimulation settings were not significantly different, apart from higher frequency in the baseline program than CBP (*p* = 0.01) or ABP (*p* = 0.003). Time spent in a program was not significantly different (CBP: 86.1 ± 29.82%, ABP: 88.6 ± 29.0%). Programing time was significantly shorter (*p* = 0.039) with ABP (19.78 ± 5.86 min) than CBP (45.22 ± 18.32).

**Conclusion:** Image-guided DBS programming in PD patients drastically reduces programming time without compromising symptom control and patient satisfaction in this small feasibility trial.

## Introduction

Deep brain stimulation (DBS) of the basal ganglia circuitry is a well-established treatment for a variety of movement disorders. Until recently, DBS programming was mainly based on clinical response testing and identification of one or several electrodes with the best motor symptom control and highest adverse effect threshold (1, 2). This process is called the *monopolar review* and still represents the gold standard in DBS programming. However, it is a complex and time-consuming task that relies on a high level of expertise and training (3) and subjects patients to a long testing procedure.

Rapidly-evolving DBS technology that has expanded the parameter space (e.g., using segmented leads) has increased the complexity of programming and prompted a search for alternative programming algorithms to streamline the programming process. Among those proposed are imaging- and electrophysiology-guided approaches (4–6).

Imaging-guided programming options require patient-specific neuroanatomical information of the target structures in the basal ganglia, combined with precise information regarding the postoperative lead location and rotation (7). Dedicated computer algorithms and software summarize this complex information in a concise graphical simulation that visualizes the leads and their individual contacts in relation to the target structures. Currently, several software solutions are available, with varying degrees of automatization and necessary interaction. These options are valuable for two main reasons: a possible reduction in programming time and a more standardized approach that could potentially reduce inter-rater variability. An analysis by Pourfar et al., showed that dedicated planning software is considerably less time-consuming than monopolar review, but leads to similar stimulation settings and motor improvements in the acute setting (8). These findings were confirmed in a small prospective pilot trial by Pavese et al., which compared the efficacy of software- and clinically-derived DBS stimulation settings in an acute cross-over approach (9).

To date, no study has been conducted to evaluate whether this novel approach works in a chronic setting and could thus be implemented in everyday patient care. Therefore, we conducted a randomized, controlled, crossover study in 10 patients to assess the feasibility of anatomical-based (image-guided) programming (ABP) in a 4 week setup, and to compare outcomes with clinical-based programming (CBP).

## Methods

### Study Design

We designed the study as an 8-week, randomized, double-blind, controlled crossover study. Inclusion criteria were the diagnosis of idiopathic bradykinetic Parkinson's syndrome, presence of a DBS stimulation system with bilateral directional electrodes in the STN with correct lead placement (at least one contact in the dorsolateral part of the STN or contacting it at least tangentially), chronic STN-DBS (>3 months), a >30% improvement in the MDS-UPDRS III score through DBS alone (Δ StimON-MedOFF/StimOFF-MedOFF), a DBS system with Cartesia™ electrodes and a Vercise PC™ or Gevia™ implantable pulse generator (IPG) (Boston Scientific, Marlborough, USA), and written consent to participate in the study. The study protocol was approved beforehand by the local ethics committee (160/18-sc).

Patients were randomly assigned in a 1:1 ratio (by drawing lots) to receive either CBP (following a directional monopolar review) or ABP (choosing active contacts based on the visualization of the electrode location and rotation in relation to the STN as a target structure). Patients and treating physicians were unaware of the group assignment. The physician responsible for activating the program was otherwise not involved in patient care or clinical assessment. All physicians participating in this study were dedicated experts in DBS therapy (5–10 years of experience in this field) and each took only one role per patient (ABP programmer or CBP programmer). The BP was defined as the program active at the baseline visit and derived by our local standard of care, i.e., a combination of initially image-based programming, clinically refined by monopolar review at 3 months postoperative, then followed by step-wise amplitude refinements at subsequent visits. The BP could be reactivated by the patient in case of severe side effects or loss of clinical efficacy not compensated by increases in amplitude. The main function of tracking the BP was to identify patients responding to DBS and to exclude non-responders from the study. By including the BP, we can exclude the possibility that an equivalent clinical outcome between CBP and ABP is due only to inferior CBP, because then CBP would also be inferior to BP.

At the beginning and at both follow-up visits (four and 8 weeks), the patients were evaluated by the physicians regarding their general well-being, the effectiveness of stimulation, side effects, and compliance with the stimulation. The neurological examination was recorded on video and later evaluated by two movement disorder experts and certified MDS-UPDRS III raters who were not otherwise associated with the study and blinded about the group the videos belonged to. For MedOFF, patients were instructed to pause extended-release dopamine agonists at least 3 days before the respective study visits, and other dopaminergic medication >12 h before the visits. In case of severe hypokinesia or painful rigidity, soluble levodopa preparations were prescribed as a rescue medication. StimOFF required a >30 min washout phase.

**Figure 1:**
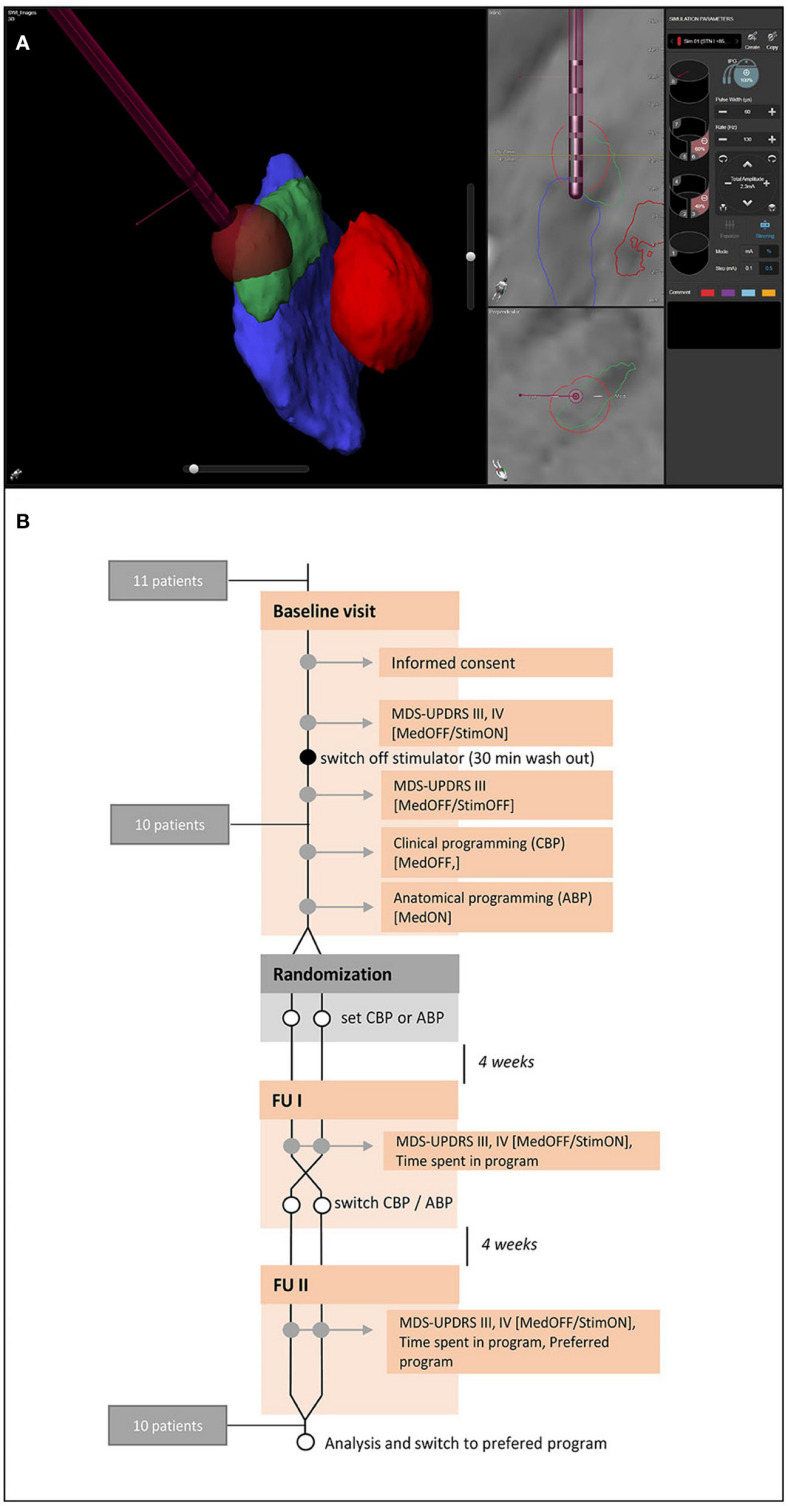
Anatomical visualization and study protocol. **(A)** Example of a visualization of the relevant nuclear structures (red, red nucleus; green, subthalamic nucleus, blue, substantia nigra) and the deep brain stimulation electrode with GuideXT. **(B)** Study protocol.

Patients were videotaped following a standardized protocol at baseline and at both follow-ups (4 and 8 weeks). Baseline measurements included motor ratings (MDS-UPDRS III) in MedOFF/StimOFF and MedOFF/StimON (the authorization to utilize the MDS-UPDRS scales was obtained from the International Parkinson's and Movement Disorder Society). Motor complications were assessed by MDS-UPDRS IV. The follow-up protocol included rating of MDS-UPDRS III in MedOFF/StimON and MDS-UPDRS IV. Any side effects or changes in medication reported by the patients were documented. Time spent in an individual program was evaluated using the stimulation log files. At the end of the study, patients were asked (without unblinding) their personal preference for one of the different programs or whether they preferred their old program (Baseline). An overview of the study protocol can be found in Figure 1.

### Directional Monopolar Review for CBP

The CBP was performed by a DBS expert as a monopolar review session in MedOFF condition for both hemispheres. To derive the best clinical-guided DBS settings, first the four individual contact levels were evaluated non-directionally for effect thresholds (complete or near complete relief of rigidity of the contralateral upper limb) and adverse effects thresholds by a stepwise increase in amplitude of 0.5 mA and then narrowed down to 0.1 mA steps. If a contact level with directional contacts (the middle two) proved to be the most effective, the directional contacts of that level were tested individually or in combination in the same way. The most effective contact or contact-combination was chosen for the final settings. Time needed for this procedure was evaluated.

The actual programming of the DBS system was performed by a DBS expert not involved in the study outcome evaluation. The choice of contacts derived by the clinical testing was programmed and the amplitude set to 0.5 mA below the clinically-tested side-effect threshold. For frequency and pulse width, we started with standard settings (130 Hz, 60 μs) and only adapted these if side effects or insufficient symptom control demanded higher/lower current injection density. All patients were treated with constant-current stimulation. Only monopolar and cathodic settings were employed.

### Anatomical Software-Based Programming

Preoperative T3 MRI scans performed under general anesthesia (T1-MPRAGE sagittal 1 mm, T2-TSE axial 2 mm, TS susceptibility-weighted image (SWI) axial isotropic 1.15 mm) and postoperative rotation fluoroscopies with flat-detector computed tomography (CT) were available for all patients included in this study. The imaging series were imported into the *Brainlab Elements* software suite (Brainlab, Munich, Germany). Images were fused by an automatic software algorithm and the accuracy visually verified. The STN, substantia nigra and nucleus ruber were segmented automatically by the software and (if deemed necessary) corrected by hand following either the SWI or T2 image series. Electrode location in depth and laterality was identified with the flat-detector CT. The lead rotation angles were determined and calculated by the *iron sight* method, based on the artifacts resulting from the overlay of small spaces between the directional contacts (10). With the help of the *GuideXT* module in the *Brainlab Elements* suite, the single contacts or contact combinations facing in direction of the dorsolateral subthalamic nucleus were identified (Figure 1). Programming the stimulator was performed in MedON by a clinician not involved into the clinical evaluation process. The projected contact settings together with a standard setting (130 Hz, 60 μs) were programmed, and the amplitude was set 0.5 mA below the side-effect threshold. As for the CBP, standard values for pulse width and frequency were only changed if side effects (e.g., dysarthria) arose or tremor control appeared suboptimal. Since the visualization software provides no other options, only monopolar settings were employed. The time that elapsed from loading the image series to printing the anatomical plan was recorded and, together with the time to adjust on the patient, was recorded as the total programming time of the ABP. For ABP programming, only imaging that was already performed in the routine implantation and post-op control procedure was used, so its performance was not included in the programming time.

### Calculation of Current Consumption

All patients had an impulse generator (IPG) equipped with *multiple independent current control* (MICC); therefore, current consumption (I_MICC_) could be calculated using equations 1 and 2 (11).


(1) $$I_{MICC}=I_{\text{overhead}}\left(f\right)+\left(\sum_{i=1}^{N}\;I_{Ei}\;*\;PW\;*\;f\;*\;\frac{V_{\text{max }}}{V_{\text{bat }}}\right)$$

Where:

I_MICC_: Current draw from battery

I_overhead_(f): Frequency-dependent IPG overhead current

N: Number of activated electrodes

I_Ei_: Pulse amplitude for electrode i

PW: Pulse width

f: Pulse frequency

V_max_: Maximum voltage for the activated electrodes

V_bat_: Battery voltage


(2) $$\begin{array}{r} V_{\max}=\max\left\{\left(I_{Ei}\;*\;Z_{Ei}\right):i=1..N\right\} \end{array}$$

Where:

Vmax: Maximum voltage for the activated electrodes

I_Ei_: Pulse amplitude for electrode i

Z_Ei_: Impedance of electrode i

N: Number of activated electrodes

Since the manufacturer does not disclose exact energy use calculations for the overhead current draw (which accounts for 2–5% of the total current draw), the overhead current was necessarily excluded from the calculation [equation (3)]:


(3) $$I_{MICC}=\left(\sum_{i=1}^{N}\;I_{Ei}\;*\;PW\;*\;f\;*\;\frac{V_{\text{max }}}{V_{bat\;}}\right)$$

As depicted above, current draw depends on the battery voltage. To make settings comparable, a common battery voltage of 2.8 V was assumed [equation (4)].


(4) $$I_{MICC}=\left(\sum_{i=1}^{N}\;I_{Ei}\;*\;PW\;*\;f\;*\;\frac{V_{\text{max }}}{2.8\;V_{\;}}\right)$$

The current draw for the left and right hemisphere was calculated individually and added up for total current draw of each of the three different programs of every patient.

### Visualization and Evaluation of the Volume of the Electrostatic Field (VEsF) of Individual Programs

The *GuideXT* module in the *Brainlab Elements* suite was used to create models of the VEsF using the stimulation settings of each group (three models for each patient: BP, CBP, ABP) (12). The patient-specific anatomical models (including the AC/PC line) and the VEsF models were exported overlayed on a 1-mm isotropic T1-MPRAGE DICOM series into a custom-built visualization tool (termed “Arena”) running in the MATLAB (MathWorks, Natick, USA) environment. The objects were normalized to the MNI2009b space (SPM12), aligned relative to the same anatomy, and mirrored to one side (right). The COG of the VEsFs were grouped (BP, CBP, ABP) and displayed in relation to the anatomy [on a normative 7T-FLASH-sequence with isotropic 100 μm resolution (13)].

### Statistical Analyses

All statistical analyses were performed with Graphpad Prism 8 (GraphPad Software, San Diego, USA). The Shapiro-Wilk test was employed for normality testing of datasets. The time spent for testing vs. planning was compared by the Wilcoxon-signed-rank test. Absolute MDS-UPDRS III values and the MDS-UPDRS III values relative to MedOFF/StimOFF for the three different groups were compared by a repeated measurements one-way ANOVA. MDS-UPDRS IV values, MDS-UPDRS IV subitem 1 values, mean stimulation amplitudes, frequencies, and pulse widths were compared by a Friedman test. Comparisons of the current draw of individual programs, VEsF volumes, and VEsF overlaps were performed by repeated measurements one-way ANOVAs. Comparison of the X-, Y-, and Z-coordinates of the individual programs was performed by a two-way ANOVA with multiple comparisons. Non-inferiority analysis was performed based on the absolute MDS-UPDRS III values with the help of an online calculator (Sealed Envelope Ltd. 2020. Available from: https://www.sealedenvelope.com/simple-randomiser/v1/) with a significance level of (alpha) 5%, a power (1- beta) of 90%, a clinical non-inferiority limit for a change >5 points (14), and a standard deviation of 7.02 [for the details of the formula see (15)]. Where appropriate, results are presented as means ± standard error of the means (SEM).

## Results

### Patient Data

Eleven patients were screened (seven men and four women). One patient was excluded due to an insufficient DBS response during the screening. The remaining 10 patients successfully completed all three visits. For demographic data, see Supplementary Table 1.

### Clinical Measures

#### Programming Time

**Figure 2:**
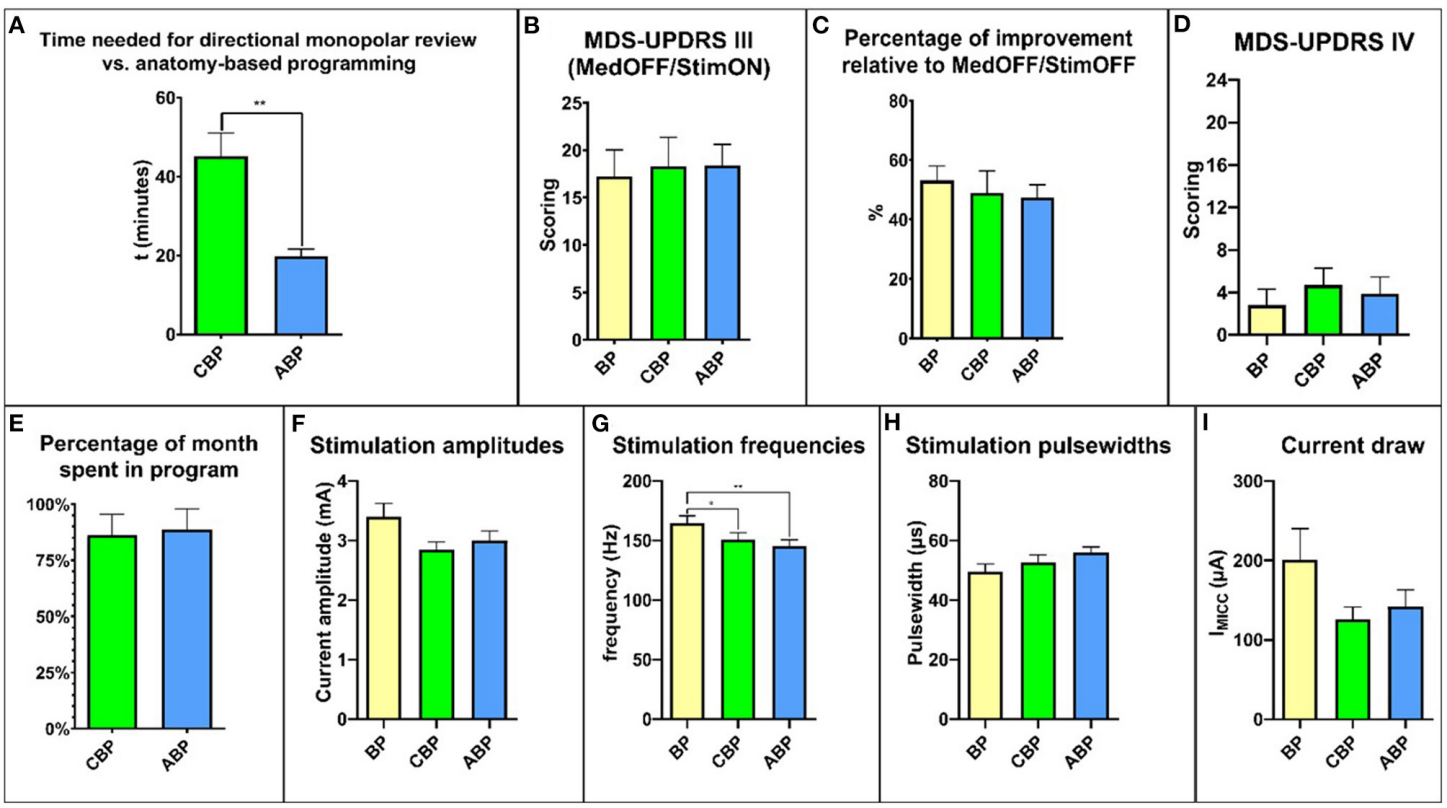
Overview of the relevant study results. **(A)** time needed for programming. **(B)** MDS-UPDRS III scores. **(C)** percent improvement relative to MedOFF/StimOFF. **(D)** MDS-UPDRS IV. **(E)** time spent in the program during the respective month of the study. **(F)** stimulation amplitudes. **(G)** stimulation frequencies. **(H)** stimulation pulse widths. **(I)** current draw. MDS-UPDRS, Movement Disorders Society Unified Parkinson's Disease Rating Scale.

There was a significant difference in the average programming time, with a mean of 45.20 ± 5.79 min for CBP and 19.78 ± 1.85 min for ABP (*p* = 0.039; Figure 2A).

#### Motor Symptom Control

According to the inclusion criteria, all patients enrolled showed an improvement of >30% in the motor symptom score (Movement Disorder Society Unified Parkinson's Disease Rating Scale (MDS-UPDRS) III) with DBS (StimON/MedOFF vs. StimOFF/MedOFF) for the baseline program (BP) (53.13 ± 14.53%). At baseline, the MDS-UPDRS III score MedOFF/StimOFF was 35.67 ± 4.57 points and MedOFF/StimON was 17.22 ± 2.81 points.

After 4 weeks, the mean MDS-UPDRS III score MedOFF/StimON was 18.27 ± 3.07 points with CBP and 18.37 ± 2.22 with ABP (Figure 2B). The relative reduction in motor symptoms compared to StimOFF/MedOFF was 48.94 ± 7.39% with CBP and 47.46 ± 4.24% with ABP (*p* = 0.39) (Figure 2C).

#### Motor Symptom Complications

The total MDS-UPDRS IV score did not differ significantly with CBP (4.1 ± 1.5; *p* = 0.35) or ABP (3.9 ± 1.5; *p* > 0.99) compared with BP (2.8 ± 1.5) (Figure 2D). There was no significant difference in the “duration of dyskinesias” subscore (item 4.1) (BP: 0.8 ± 0.3; CBP: 0.7 ± 0.3; ABP:1 ± 0.4; *p* > 0.99) (Supplementary Figure 1).

#### Programming Characteristics

For a comprehensive overview, see Supplementary Table 1. The time spent in a particular program, expressed as a percentage between the follow-up visits, was 86.1 ± 9.42% for CBP and 88.6 ± 9.1% for ABP (*p* = 0.99; Figure 2E). The reasons for changing back to the BP were mainly dysarthria and gait difficulties (Supplementary Table 1). Four patients preferred BP, three CBP and three ABP.

#### Future Sample Size Calculation

From the group mean data and variances, we estimate that a sufficiently-powered study proving non-inferiority of image-guided programming compared to clinical-based programming using the UPDRS motor score as primary variable would need a total of 44 patients. Future studies, of a comparative nature, could be planned on this basis.

### Stimulation Settings and Current Consumption

The mean amplitude was 3.36 ± 0.23 mA for the BP, 2.84 ± 0.13 mA for CBP, and 3.00 ± 0.16 mA for ABP (Figure 2F). The mean stimulation frequency was 164.5 ± 6.3 Hz for the BP, 150.8 ± 5.8 Hz for CBP, and 145.3 ± 5.3 Hz for ABP (Figure 2G). The mean pulse width was 49.55 ± 2.58 ms for the BP, 52.73 ± 2.38 ms for CBP, and 55.9 ± 1.93 ms for ABP (Figure 2H).

The stimulation settings did not differ significantly regarding amplitude (*p* = 0.35) or pulse width (*p* = 0.053), but the mean stimulation frequency was significantly higher for the BP than CBP (*p* = 0.01) and ABP (*p* = 0.003). The mean current draw was 200.6 ± 39.52 μA for the BP, 125.6 ± 16.05 μA for CBP, and 141.7 ± 21.19 μA for ABP (Figure 2I). There were no significant differences between the programs (BP vs. CBP: *p* = 0.77; BP vs. ABP: *p* = 0.98; ABP vs. CBP: *p* = 0.65).

### Visualization and Evaluation of the VEsFs of Individual Programs

**Figure 3:**
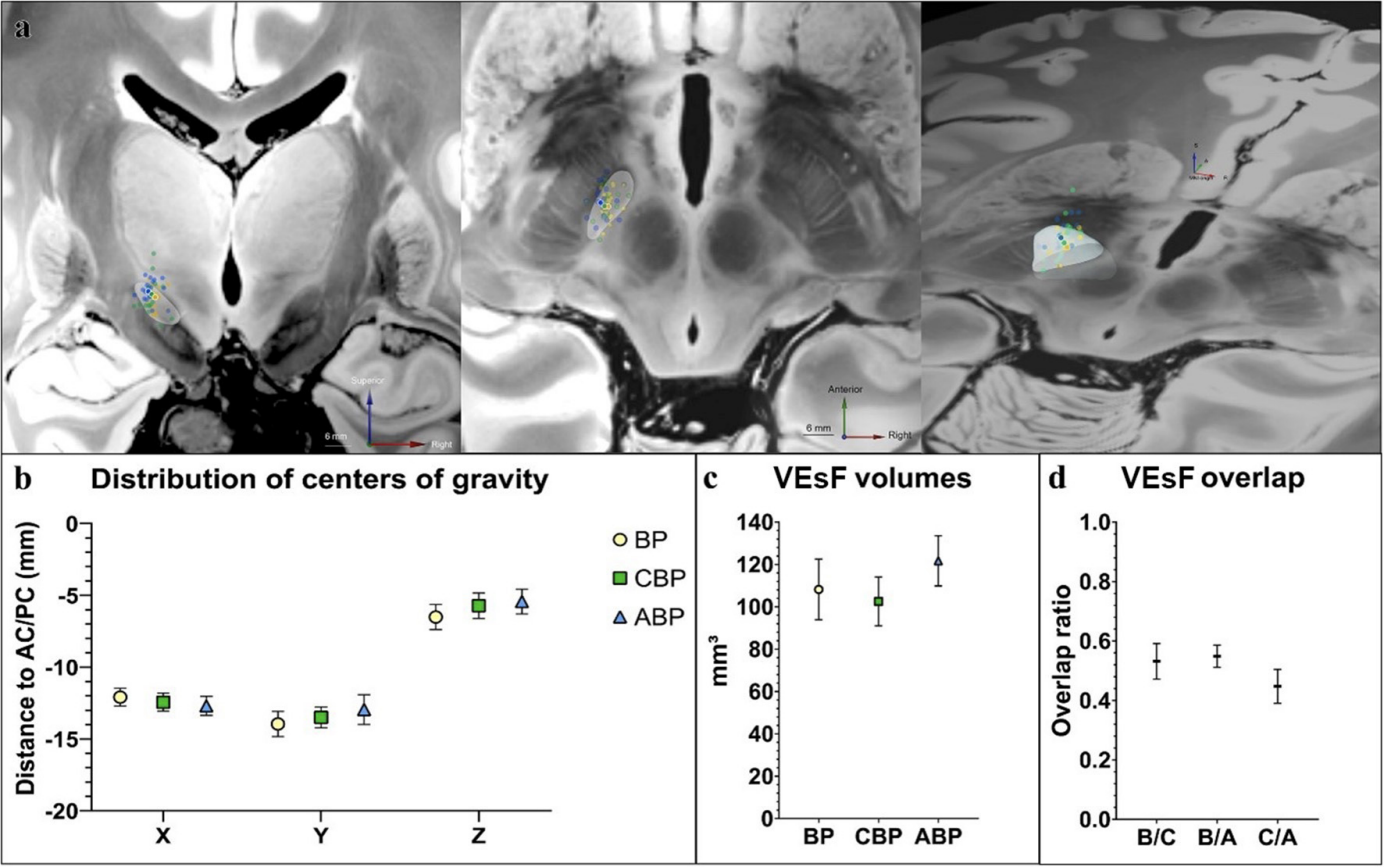
Comparison of stimulation programs by VEsF modeling. **(a)** Visualization of the COG of all VEsFs displayed on a coronal plane, axial plane, and as oblique bird's eye view on a normalized 7T-FLASH-sequence (yellow dots, BP VEsFs; green dots, CBP VEsFs; blue dots, ABP VEsFs, the mean COG of each group is highlighted with a white outline). **(b)** overview of the distribution of COG. **(c)** VEsFs volumes. **(d)** VEsF overlap between individual groups. ABP, anatomical-based programming; BP, baseline program; CBP, clinical-based programming; COG, centers of gravity; VEsF, Volumes of the electrostatic field.

The distribution of centers of gravity (COG) of “volume of the electrostatic field” (VEsF) sorted by group was not significantly different according to the MNI-x/y/z-coordinates of the BP (−12.00/−13.89/−6.16), CBP (−12.46/−13.82/−5.68), and ABP (−12.99/−13.40/−5.29) – BP vs. CPB: x-axis *p* = 0.52, y-axis *p* = 0.92, z-axis *p* = 0.53; CBP vs. APB: x-axis *p* = 0.49, y-axis *p* = 0.53, z-axis *p* = 0.49; BP vs. APB: x-axis *p* = 0.19, y-axis *p* = 0.48, z-axis *p* = 0.09 (Figures 3a,b).

The VEsF of the individual groups did not differ significantly between BP (108.20 ± 13.94 mm^3^), CBP (102.55 ± 11.22 mm^3^), and ABP (121.7 ± 11.52 mm^3^) (*p* = 0.22; Figure 3c). Overlap of individual patient's VEsFs between programming groups was generally low, with mean values of 0.53 ± 0.06 for BP/CBP, 0.55 ± 0.04 for BP/ABP, and 0.45 ± 0.06 for CBP/ABP (Figure 3d).

## Discussion

This is the first study to examine the clinical effects of stimulation settings derived using imaging-based DBS programming based on individual patient anatomy in a prospective, chronic, and double-blinded setting.

The results presented here suggest that imaging-based programming can achieve i) stable symptom control in the chronic course, ii) requires significantly shorter programming times than the previous gold standard and is thus faster to deploy, iii) indicatescomparable rates of side effects and power consumption.

The quasi-identical symptom control between this new technique and the gold standard is remarkable, but cannot be conclusively characterized in this feasibility study, as the study was not adequately powered and thus, a type I statistical error cannot be ruled out. So we advise the reader to keep in mind that these are preliminary data that need replication in larger trials to get translated into clinical practice However, these results provide a very solid basis for planning and paving larger, multi-center studies to explore this potential more thoroughly.

### Clinical Outcome

For the PD patient, the primary goal of DBS is to provide consistently solid symptom control without compromise by side effects, fluctuations, or repeated reprogramming sessions (16). For anatomically guided programming to be perceived as acceptable to the patient, especially in the long-term course, at least these aspects must perform comparably to the clinically best possible programming.

A surrogate parameter for program acceptability was the high duration of time spent in program at 85% of study duration. Since all patients were trained in the use of the DBS remote control and were instructed to change the program after appropriate consultation if they were not satisfied with the performance, such a long duration of time spent in the program indicates a general satisfaction with the proposed program.

This overall high satisfaction can be well-explained by the comparably good results in control of motor symptoms, occurrence of side effects and motor fluctuations of both programs. Both programs (the anatomically based and the clinically based programming) were perceived by the patients as equivalent and equally good, so that the question of the feasibility of this novel method in terms of effectiveness can clearly be affirmed.

The study was not primarily designed as a non-inferiority analysis, but to provide these effect sizes in addition to the feasibility analysis. Based on our findings, it can be assumed that a randomized controlled trial needs group sizes of about 40–50 patients, i.e., a moderate study size, which can be performed well in a multicenter setting.

In terms of technical feasibility and applicability in clinical practice, results of previous pilot studies indicated a reduction in programming time with the novel programming approach (9). In this study, this effect was not only reproduced, but was unequally more pronounced. Until a few years ago, commercially available electrodes were quadripolar, so that four contacts had to be tested out during clinical programming (CBP). The directional electrodes used in this study are octopolar, with two rings of three contacts each and two single contacts. In clinical practice, this allows for more differentiated programming, but the new variety of contacts and resulting combinations drastically increases programming time if all contacts are to be tested as before.

Thus, while more modern electrodes significantly increase the complexity of testing for CBP, only a few additional clicks in the software are required for ABP, which was impressively demonstrated in this study by the drastic reduction in programming time: at 45 min, CBP took more than twice as long as ABP at 19 min.

### Anatomical DBS Programming Approach

Our approach to ABP aims to select a single electrode or a combination of electrodes in closest proximity and direction toward the dorsolateral aspect of the subthalamic nucleus (STN). The stimulation amplitude is still determined clinically by defining the side effect threshold and aiming at 0.5 mA below. This approach does not represent *in silico* programming in the strict sense. The GuideXT software includes an option to model the volume of the electrostatic field (VEsF) (12). The use of such models is controversial, in part because these models assume a uniform distribution of axons with specific conduction properties around the electrode, which does not truly reflect the complex geometry and heterogeneity of fiber tracts in the stimulated brain region (17).

Discussions about the possible uses of corresponding models are far from over, but what is certain is that they are still under development. Accordingly, we have decided to completely avoid these models for patient care and used anatomic information only. The study was followed by *post-hoc* analyses of electrode locations and electric field propagation, where these models are again indispensable.

Our scenario still required a DBS programmer with basic training to set the ABP. However, since the otherwise complex programming was reduced to a simplified check of the adverse event thresholds for a given contact selection, the required know-how and training is substantially lower than in the current clinical practice (which corresponds to the CBP approach). Determining the amplitude setting using the above-mentioned approach does not reflect our usual clinical standard of care, in which the current is gradually increased for longer timespans. This might limit the comparability with the baseline program but represents the ability to define settings in a single programming session. Furthermore, there might be a certain risk of overstimulation. We therefore specifically determined the dyskinesia subscale of the MDS-UPDRS IV (item 4.1). Despite a small trend in higher values for ABP, CBP showed lower values than the BP. We therefore conclude that the study approach did not result in a clinically-relevant overstimulation.

In terms of patient comfort, ABP is not dependent on patients remaining in the MedOFF for testing. This is different to standard clinical care where DBS programming is usually performed in the MedOFF state. Although this assessment was not part of the study, in our experience we would expect patients to appreciate not having to undergo this uncomfortable and sometimes even painful procedure.

### Volumes of the Electrostatic Field (VEsF) and Energy Consumption

VEsF models were used only *post-hoc* to analyze stimulation at the group level and not for patient programming. We performed two analyses on the comparison of VEsFs between groups, which are presented in detail in Supplementary Figure 2; Supplementary Table 3. In summary, all groups show a commonly stimulated area in the dorsolateral STN (Supplementary Figure 2C), which could explain the good effect therapeutic efficacy of all groups.

Methods like this have been repeatedly tested to identify a sweet spot of subthalamic neurostimulation. However, similar conclusions should not be drawn from the data of our study because the study was not designed to answer this question and the methodology used was accordingly not tailored to do so. A one-to-one comparison of the VEsF shows a wide range of overlap, with Dice coefficients of all combinations used as a measure of the overlap of the VEsF (Supplementary Table 3).

In accordance with the similar size of the VEsF, current draw between individual programs was comparable. This excludes the possibility that the same clinical efficacy was merely achieved by an increase in amplitude for an otherwise suboptimal contact combination.

## Conclusion

Imaging-guided STN-DBS for Parkinson's disease patients is technically feasible, safe, easy to use in daily clinical practice, provides good patient satisfaction as well as symptom control in chronic DBS, while enabling a drastic reduction in programming time.

While some results can already be concluded from this small study (esp. the significantly reduced programming time), others now allow the paving of comparative studies between this novel technique and the gold standard of clinical programming (effect sizes of motor symptom control, side effects, and fluctuations).

## Data Availability Statement

The original contributions presented in the study are included in the article/Supplementary Material, further inquiries can be directed to the corresponding author/s.

## Ethics Statement

The studies involving human participants were reviewed and approved by Ethik-Kommission der Universität Würzburg. The patients/participants provided their written informed consent to participate in this study.

## Author Contributions

Conception and design of the study was done by FL, FS, JR, MR, and PC. Data acquisition was performed by FL, TM, TO, GB, and PC. Article drafting was performed by FL, and PC. FS, TO, CM, and JV: contributed to critically reading and revising the manuscript. All authors were involved in analyzing and interpretation of the data and gave final approval to all manuscript versions submitted.

## Funding

This publication was supported by the Open Access Publication Fund of the University of Wuerzburg.

## Conflict of Interest

FL, GB, FS, TO, MR, JV, CM, and PC have received commercial funding prior to this study. The remaining authors declare that the research was conducted in the absence of any commercial or financial relationships that could be construed as a potential conflict of interest. All authors declare that this study has not received any industrial funding.

## Publisher's Note

All claims expressed in this article are solely those of the authors and do not necessarily represent those of their affiliated organizations, or those of the publisher, the editors and the reviewers. Any product that may be evaluated in this article, or claim that may be made by its manufacturer, is not guaranteed or endorsed by the publisher.
