# Supplementary material for: Reduced Programming Time and Strong Symptom Control Even in Chronic Course Through Imaging-Based DBS Programming
Source: Front Neurol. 2021 Nov 8;12:785529. doi: 10.3389/fneur.2021.785529 (PMC8606823; doi:10.3389/fneur.2021.785529)
Supplement: Supplementary file 1 [file Data_Sheet_1.docx]

Supplemental figure 1 - Duration of Dyskinesias:

Comparison of MDS-UPDRS IV subscore 4.1 reveals no significant differences between the individual programs.

Supplemental figure 2 – Overview of VEsF. A) shows the wide spread of color-coded individual VEsF in the area of the dorso-lateral STN. B) indicates a mean VEsF for each group (with the the mean size of all individual VEsF = 99mm3). C) shows the commonly stimulated volumes of the groups are closer together than expected, resulting in a commonly stimulated volume of all groups. This red cloud thus represents the largest intersection of all VEsF in this study


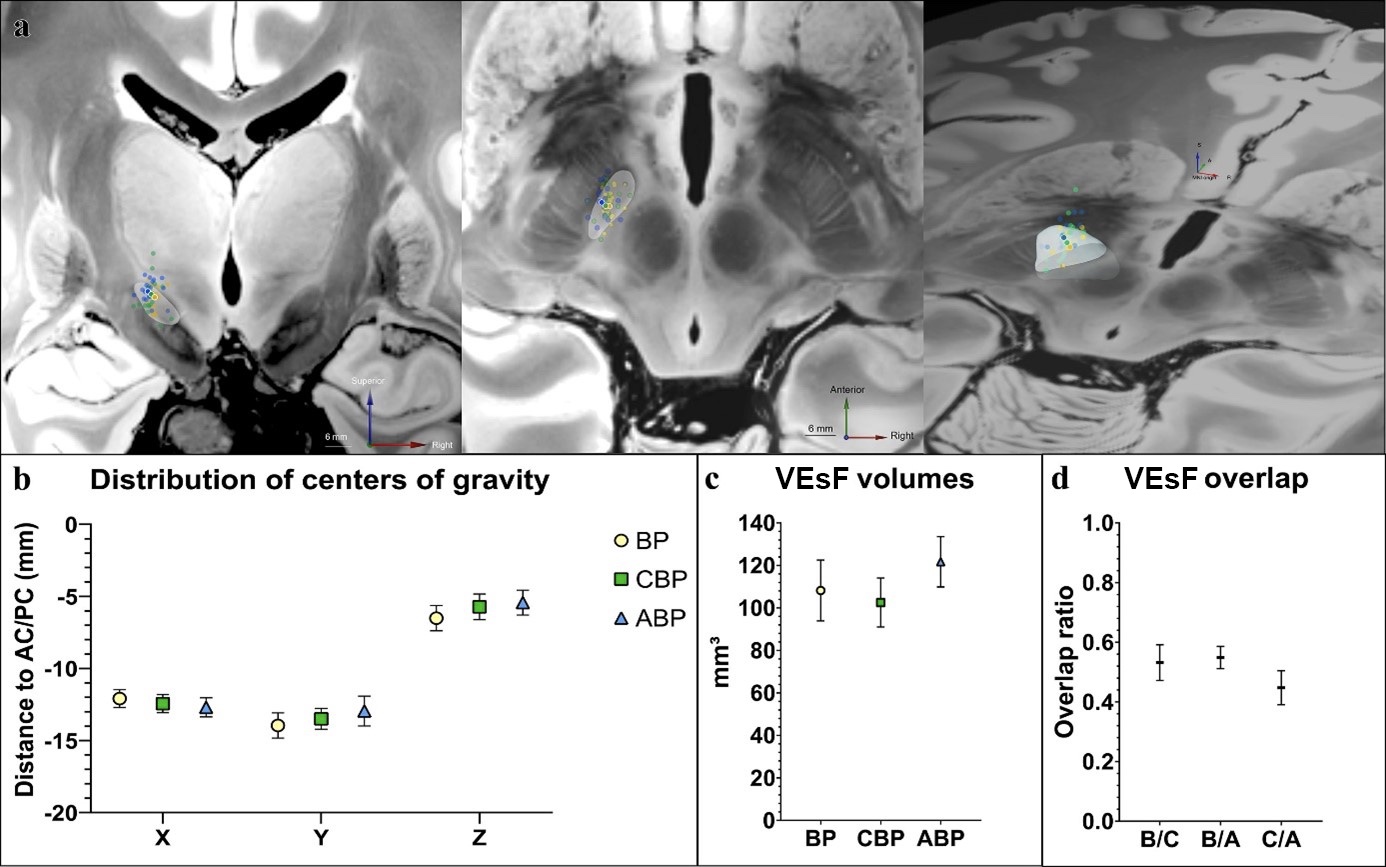


Supplementary Table 1. Demographic characteristics of patients, overview of stimulation settings, motor and non-motor scores, side effects, programming time, and patient preference.

| **Patient**  **Age/Sex**  **PP** | **Disease duration (yrs)/Years since implantation (yrs)** | **MDS-UPDRS III MedOFF-StimON** | **Relative reduction**  **MDS-UPDRS III** | **MDS-UPDRS IV** | **Frequency**  **(Hz)**  **right/left** | **Amplitude**  **(mA)**  **right/left** | **Pulse width**  **(µs)**  **right/left** | **Current draw**  **(µA)** | **Programming time**  **(min)** | **Side effects leading to a discontinuation of programs** |
| --- | --- | --- | --- | --- | --- | --- | --- | --- | --- | --- |
| **68/M**  BP  CPB  ABP | 11/4 | 33.67  28.33  23.33 | 47.4  55.7  63.5 | 0  7  0 | 185/185  185/185  130/130 | 4.7/4.2  2.5/3.0  2.0/3.5 | 30/40  40/40  60/60 | 253.44  89.83  46.51 | 27  35 | IMSC |
| **66/M**  BP  CPB  ABP | 14/4 | 10.00  8.00  12.33 | 55.8  64.7  45.5 | 1  0  0 | 185/185  179/179  179/179 | 4.0/4.0  2.6/3.5  3.8/4.4 | 40/30  60/60  60/60 | 128.10  191.79  267.34 | 45  18 |  |
| **57/M**  BP  CPB  ABP | 15/14 | 20.00  15.67  25.00 | 62.9  70.9  53.7 | 2  5  6 | 130/130  130/130  130/130 | 5.0/4.0  3.0/3.0  3.0/3.0 | 60/60  60/60  60/60 | 416.28  111.69  152.90 | 60  21.5 |  |
| **67/F**  BP  CPB  ABP | 15/2 | 2.00  3.33  5.67 | 88.9  81.5  68.5 | 1  1  11 | 185/231  185/185  185/185 | 1.7/3.4  2.0/3.5  2.0/3.8 | 40/40  40/60  40/40 | 97.41  127.36  118.82 | 90  17.4 |  |
| **58/M**  BP  CPB  ABP | 19/6 | 21.00  29.67  23.00 | 30.0  1.1  23.3 | 2  7  0 | 159/159  130/130  130/130 | 5.1/3.9  4.0/3.0  3.0/3.5 | 40/30  60/60  60/60 | 334.45  132.81  118.31 | 40  13.45 | Dysarthria  Dysarthria |
| **52/F**  BP  CPB  ABP | 17/5 | 13.33  18.33  13.33 | 51.8  33.7  51.9 | 0  5  1 | 179/179  179/179  179/179 | 4.0/3.9  3.0/3.9  3.8/4.0 | 60/60  60/60  60/60 | 250.51  182.68  152.17 | 35  20 |  |
| **71/M**  BP  CPB  ABP | 18/1 | 19.33  21.33  26.00 | 55.0  50.4  39.5 | 4  1  6 | 154/154  130/130  130/130 | 2.5/2.0  2.6/1.6  2.5/2.0 | 40/60  40/60  40/60 | 73.95  40.42  54.79 | 45  15 |  |
| **52/M**  BP  CPB  ABP | 11/2 | 11.33  9.00  12.00 | 50  60.87  47.8 | 1  1  0 | 185/185  130/130  130/130 | 4.0/2.0  3.7/2.7  3.4/3.7 | 60/60  60/60  60/60 | 291.98  165.44  207.55 | 45  20 |  |
| **69/F**  BP  CPB  ABP | 16/2 | 26.00  32.00  24.00 | 43.4  30.4  47.8 | 16  17  13 | 130/130  130/130  130/130 | 2.0/2.0  2.5/2.5  3.0/3.0 | 60/60  40/60  60/60 | 53.62  59.61  182.82 | 30  19.45 | Gait problems  Dysarthria |
| **64/M**  BP  CPB  ABP | 15/1 | 15.33  17.00  19.00 | 45.8  40  32.9 | 1  3  2 | 130/130  130/130  130/130 | 2.3/2.0  2.5/2.5  2.3/2.3 | 60/60  60/60  60/60 | 105.96  153.99  115.99 | 35  18 | Gait problems |
| **Mean**  BP  CPB  ABP | 16.1 ± 0.8 /  5.1 ± 1.2 | 17.2 ± 2.6  18.2 ± 2.9  18.3 ± 2.1 | 53.1 ± 4.5  48.9 ± 7.0  47.4 ± 4.0 | 2.8 ± 1.5  4.1 ± 1.5  3.9 ± 1.5 | **164.5 ± 6.3 †**  150.8 ± 5.8  145.3 ± 5.3 | 3.3 ± 0.2  2.8 ± 0.1  3.0 ± 0.1 | 49.55 ± 2.5  52.73 ± 2.3  55.91 ± 1.9 | 200.6 ± 39.5  125.6 ± 16.0  141.7 ± 21.1 | 45.2 ± 5.7  **19.78 ± 1.8 †** |  |

**†** Significantly different, p<0.05. IMSC, insufficient motor symptom control; MDS-UPDRS, Movement Disorders Society Unified Parkinson’s Disease Rating Scale; PP, patient preference.

Supplementary table 2. Overview of active contacts

| **Patient**  **Age/Sex**  **PP** | **Active contact**  **left STN** | **Active contact**  **right STN** |
| --- | --- | --- |
| **68/M**  BP  CPB  ABP | C+, 7-(100%)  C+, 2-(100%)  C+, 8-(50%), 7-(25%), 5- (25%) | C+,13-(70%), 16-(30%)  C+,16-(100%)  C+, 16-(30%), 15-(35%), 13- (35%) |
| **66/M**  BP  CPB  ABP | C+,2-(20%),4-(20%),5-(30%),7-(30%)  C+, 4-(100%)  C+,6-(20%),7-(20%),8-(60%) | C+,10-(25%), 11-(25%), 13-(25%), 14-(25%)  C+,13-(100%)  C+,16-(100%) |
| **57/M**  BP  CPB  ABP | 3+(50%), 6+(50%), 1-(30%),2-(18%),4-(52%)  C+, 4-(50%),7-(50%)  C+,3-(35%),4-(35%),6-(15%),7-(15%) | C+, 10-(34%),11-(34%),12-(33%)  C+, 13-(50%),15-(50%)  C+, 12-(10%),15-(90%) |
| **67/F**  BP  CPB  ABP | C+, 7-(100%)  C+, 4-(100%)  C+,2-(5%),4-(5%),5-(45%),7-(45%) | C+, 10-(20%),11-(20%),13-(30%),14-(30%)  C+, 11-(35%),12-(35%),16-(30%)  C+, 13-(28%),14-(13%),15-(59%) |
| **58/M**  BP  CPB  ABP | 3+(50%),4+(50%), 1-(100%)  C+, 8-(100%)  C+,1-(20%),2-(60%),3-(20%) | 12+, 9-(100%)  C+, 11-(100%)  C+, 9-(10%),10-(22%),12-(68%) |
| **52/F**  BP  CPB  ABP | C+, 5-(50%),6-(50%)  C+, 6-(100%)  C+,5-(35%),6-(35%), 8-(30%) | C+, 10-(25%),11-(75%)  C+, 10-(25%),12-(25%),13-(25%),15-(25%)  C+, 13-(40%),15-(40%),16-(20%) |
| **71/M**  BP  CPB  ABP | C+, 5-(12%),6-(38%), 8-(50%)  C+, 8-(100%)  C+, 8-(100%) | C+, 13-(70%),16-(30%)  C+, 13-(35%),15-(35%),16-(30%)  C+, 16-(100%) |
| **52/M**  BP  CPB  ABP | 8+,2-(40%),4-(60%)  C+, 2-(50%),5-(50%)  C+, 5-(50%),7-(50%) | C+, 13-(34%),14-(33%),15-(33%)  C+, 10-(34%),11-(33%),12-(33%)  C+, 16-(100%) |
| **69/F**  BP  CPB  ABP | C+,6-(100%)  C+, 1-(100%)  C+, 3-(50%),4-(50%) | C+, 13-(18%),14-(16%),15-(16%),16-(50%)  C+, 10-(50%),13-(50%)  C+, 14-(75%),15-(25%) |
| **64/M**  BP  CPB  ABP | C+,4-(40%),7-(40%), 8-(20%)  C+, 1-(50%),4-(50%)  C+, 7-(100%) | C+, 16-(100%)  C+, 13-(34%),14-(33%),15-(33%)  C+, 16-(100%) |
| **Mean**  BP  CPB  ABP | C+, 3-(100%)  C+, 5-(25%),6-(75%)  C+, 4-(100%) | C+, 13-(50%),15-(50%)  C+, 10-(30%),13-(70%)  C+, 12-(50%),15-(50%) |

Supplementary table 3. Dice coefficients of all VEsF against the corresponding VEsF of the same patients. A Dice coefficient of 1.00 corresponds to a complete overlap or the same VEsF. A Dice coefficient of 0.00 means no overlap at all.

| **VTA** | **Left**  **ABP** | **Left**  **CBP** | **Left**  **BP** | **Right**  **ABP** | **Right**  **CBP** | **Right**  **BP** |  |  |  |  |
| --- | --- | --- | --- | --- | --- | --- | --- | --- | --- | --- |
| ABP1 | 1 | 0,16 | 0,57 | 1 | 0,51 | 0,55 |  |  |  |  |
| CBP1 | 0,16 | 1 | 0,2 | 0,51 | 1 | 0,82 |  |  |  |  |
| BP1 | 0,57 | 0,2 | 1 | 0,55 | 0,82 | 1 |  |  |  |  |
| ABP2 | 1 | 0,27 | 0,49 | 1 | 0,59 | 0,49 |  |  |  |  |
| CBP2 | 0,27 | 1 | 0,65 | 0,59 | 1 | 0,26 |  |  |  |  |
| BP2 | 0,49 | 0,65 | 1 | 0,49 | 0,26 | 1 |  |  |  |  |
| CBP3 | 1 | 0,73 | 0,59 | 1 | 0,44 | 0,73 |  |  |  |  |
| BP3 | 0,73 | 1 | 0,54 | 0,44 | 1 | 0,51 |  |  |  |  |
| ABP3 | 0,59 | 0,54 | 1 | 0,73 | 0,51 | 1 |  |  |  |  |
| ABP4 | 1 | 0,67 | 0,72 | 1 | 0,48 | 0,33 |  |  |  |  |
| CBP4 | 0,67 | 1 | 0,89 | 0,48 | 1 | 0,61 |  |  |  |  |
| BP4 | 0,72 | 0,89 | 1 | 0,33 | 0,61 | 1 |  |  |  |  |
| ABP5 | 1 | 0 | 0,11 | 1 | 0,34 | 0,49 |  |  |  |  |
| CBP5 | 0 | 1 | 0,56 | 0,34 | 1 | 0,45 |  |  |  |  |
| BP5 | 0,11 | 0,56 | 1 | 0,49 | 0,45 | 1 |  |  |  |  |
| CBP6 | 1 | 0,74 | 0,82 | 1 | 0,68 | 0,4 |  |  |  |  |
| BP6 | 0,74 | 1 | 0,76 | 0,68 | 1 | 0,64 |  |  |  |  |
| ABP6 | 0,82 | 0,76 | 1 | 0,4 | 0,64 | 1 |  |  |  |  |
| ABP7 | 1 | 0,92 | 0,92 | 1 | 1 | 0,44 |  |  |  |  |
| CBP7 | 0,92 | 1 | 1 | 1 | 1 | 0,44 |  |  |  |  |
| BP7 | 0,92 | 1 | 1 | 0,44 | 0,44 | 1 |  |  |  |  |
| ABP8 | 1 | 0,67 | 0,65 | 1 | 0,33 | 0,05 |  |  |  |  |
| CBP8 | 0,67 | 1 | 0,47 | 0,33 | 1 | 0,36 |  |  |  |  |
| BP8 | 0,65 | 0,47 | 1 | 0,05 | 0,36 | 1 |  |  |  |  |
| ABP9 | 1 | 0,1 | 0,48 | 1 | 0,26 | 0,4 |  |  |  |  |
| CBP9 | 0,1 | 1 | 0,37 | 0,26 | 1 | 0,16 |  |  |  |  |
| BP9 | 0,48 | 0,37 | 1 | 0,4 | 0,16 | 1 |  |  |  |  |
| ABP10 | 1 | 0,23 | 0,5 | 1 | 0,67 | 0,4 |  |  |  |  |
| CBP10 | 0,23 | 1 | 0,39 | 0,67 | 1 | 0,56 |  |  |  |  |
| BP10 | 0,5 | 0,39 | 1 | 0,4 | 0,56 | 1 |  |  |  |  |
|  |  |  |  |  |  |  |  |  |  |  |
|  |  |  |  |  |  |  |  |  |  |  |
|  |  |  |  |  |  |  |  |  |  |  |
|  |  |  |  |  |  |  |  |  |  |  |
